# On Cell Loss and Selective Vulnerability of Neuronal Populations in Parkinson's Disease

**DOI:** 10.3389/fneur.2018.00455

**Published:** 2018-06-19

**Authors:** Nicolas Giguère, Samuel Burke Nanni, Louis-Eric Trudeau

**Affiliations:** CNS Research Group, Department of Pharmacology and Physiology, Department of Neurosciences, Faculty of Medicine, Université de Montréal, Montreal, QC, Canada

**Keywords:** Parkinson, vulnerability, dopamine, cell death, neurodegeneration

## Abstract

Significant advances have been made uncovering the factors that render neurons vulnerable in Parkinson's disease (PD). However, the critical pathogenic events leading to cell loss remain poorly understood, complicating the development of disease-modifying interventions. Given that the cardinal motor symptoms and pathology of PD involve the loss of dopamine (DA) neurons of the substantia nigra pars compacta (SNc), a majority of the work in the PD field has focused on this specific neuronal population. PD however, is not a disease of DA neurons exclusively: pathology, most notably in the form of Lewy bodies and neurites, has been reported in multiple regions of the central and peripheral nervous system, including for example the locus coeruleus, the dorsal raphe nucleus and the dorsal motor nucleus of the vagus. Cell and/or terminal loss of these additional nuclei is likely to contribute to some of the other symptoms of PD and, most notably to the non-motor features. However, exactly which regions show actual, well-documented, cell loss is presently unclear. In this review we will first examine the strength of the evidence describing the regions of cell loss in idiopathic PD, as well as the order in which this loss occurs. Secondly, we will discuss the neurochemical, morphological and physiological characteristics that render SNc DA neurons vulnerable, and will examine the evidence for these characteristics being shared across PD-affected neuronal populations. The insights raised by focusing on the underpinnings of the selective vulnerability of neurons in PD might be helpful to facilitate the development of new disease-modifying strategies and improve animal models of the disease.

## Introduction

Parkinson's disease (PD) was first described two centuries ago in *An essay on the shaking palsy* (1). Since then, great strides have been made in understanding the disease basics. However—as with many other neurodegenerative disorders—there is still no disease modifying treatment for PD. Unfortunately, progress has been slow, and a thorough understanding of the pathological processes has been elusive.

PD as a clinical diagnosis is characterized by the detection of significant motor deficits (including bradykinesia, resting tremor, and rigidity) due, in large part, to a loss of dopamine (DA)-containing neurons of the substantia nigra pars compacta (SNc). The SNc is a neuronal population projecting to the caudate and putamen and is critical for regulation of basal ganglia circuitry. At clinical presentation, it has been estimated that 40–60% of SNc DA neurons have already degenerated (2, 3). The clinical features of the disease are diverse and include substantial non-motor features including, autonomic and olfactory dysfunction, constipation, sleep disturbances, depression, and anxiety (4–6).

The diagnostic criteria for PD have been recently re-defined by the International Parkinson and Movement Disorder Society (MDS), with the MDS Clinical Diagnostic Criteria for Parkinson's disease [MDS-PD Criteria (7)]. A diagnosis is made when there is documented parkinsonism (defined as bradykinesia, with tremor at rest and/or rigidity), followed by the exclusion of other possible causes of parkinsonism, and with additional supporting criteria, including olfactory dysfunction or cardiac sympathetic denervation [see (7)]. The recent nature of this re-evaluation illustrates both the heterogeneity of PD expression, and the difficulties encountered in defining it.

In ≈70% of the ‘clinically typical PD cases’, the hallmark pathological finding is the presence of Lewy pathology (LP) in the SNc (4, 5)—however, LP is also found across the central, peripheral, and enteric nervous system (CNS, PNS, and ENS) (6). This includes both Lewy bodies and Lewy neurites: both similar cellular inclusions, formed predominantly of aggregated α-synuclein, but also including a large number of different molecules, proteins and organelles, such as ubiquitin, tubulin, neurofilaments, lipids, and mitochondria (8).

In considering the broad localization of LP and the origins of the various symptoms of PD, a critical point to consider is the dysfunction and loss of neurons in regions of the CNS and PNS, other than the SNc. There have been, indeed, many studies concluding that cholinergic neurons in the pedunculopontine nucleus (PPN), noradrenergic neurons of the locus coeruleus (LC), cholinergic neurons of the nucleus basalis of Meynert (NBM) and of the dorsal motor nucleus of the vagus (DMV), and serotonergic neurons of the raphe nuclei (RN) are lost in PD. The strength of the evidence for actual neuronal cell body loss in these regions is highly variable and is one of the questions addressed in the present review. The fact that the diagnostic criteria for PD have over time been refined adds another layer of complexity to the task of identifying the origin of the diverse symptoms of PD. Presently, PD is classified into either primary or secondary subtypes. Primary parkinsonism includes genetic and idiopathic forms of the disease and secondary parkinsonism includes forms induced by drugs, infections, toxins, vascular defects, brain trauma or tumors or metabolic dysfunctions. This second subtype of PD is also sometimes called atypical parkinsonism when concomitant to progressive supranuclear palsy, multiple system atrophy or corticobasal degeneration, for example.

Since pathology is likely to emerge through different processes depending of PD subtypes, and since modern classification was non-existent when a substantial part of the research literature was produced, attempting to reach clear general vision of various pathophysiological markers and their link to disease progression for each sub-type of PD presents a significant challenge. This review will primarily focus on idiopathic PD, since this category represents the large majority of cases and is likely to represent most of the subjects examined in studies where PD type was not provided.

Another main hurdle in PD research is that the chain of events that leads to the death of neurons is still not clear. The fact that pathology is thought to begin years/decades before the appearance of symptoms might, in part, explain this lack of progress.

PD has been considered to exist as either a strictly monogenetic or environmentally-triggered disease, as well as a mixture of the two. The pathological mechanisms at the core of each form have been proposed to converge in causing cellular stress secondary to mitochondrial dysfunction, perturbed proteostasis and elevated oxidative stress. A major conundrum is that at first glance, these factors alone fail to explain why PD pathology is restricted to very limited subsets of brain nuclei. Therefore, a key question is what do these PD sensitive neurons have in common and what is it about them that renders them more vulnerable compared to neurons from other brain regions?

A better understanding of the fundamental nature of cell loss and cellular dysfunction in the parkinsonian brain is required to develop critically needed, novel, therapeutic strategies. In this review, we aim to re-evaluate the evidence for cell loss in PD, then to highlight the common characteristics that could explain their selective vulnerability.

## Physiopathology of parkinson's disease

The focus on SNc DA neurons has brought significant advances in our understanding of PD pathophysiology, as well as of the signaling pathways that lead to DA neuron death. Studies using DA neuron selective toxins such as 6-OHDA and MPTP, as well as investigations of gene products mutated in familial forms of the disease (including α-synuclein, Parkin, Pink1, LRRK2, DJ-1, and GBA1), have been instrumental to better understand some of the key dysfunctional processes implicated in the disease. These include protein clearance (9–11), mitochondrial turnover (12–14), ROS management (15, 16), and inflammation (17, 18). Perturbations of these processes have been proposed to underlie distinct physiological dysfunctions in PD-vulnerable neurons (19). Nonetheless, since the first introduction of Levodopa in the 1950s and the development of deep-brain stimulation in the 1990s, increased understanding of PD pathophysiology has not yet permitted the discovery of disease-modifying therapies.

As stated previously, PD is more than just a disease of DA and the SNc. Non-motor symptoms—including a reduced sense of smell, constipation, orthostatic hypotension, sleep disturbances, depression, and anxiety—are likely to be due to impaired function and/or loss of non-DA neurons (20). There has thus been a growing interest in better understanding the implications of other regions of the CNS and PNS in the progression of PD pathology. In the early 2000s, pioneering work by Braak and colleagues defined stages in PD based on the appearance of LP in various regions of the nervous system, correlating their findings to the symptomatic progression of the disease (21–23). Most notably, LP was detected in the dorsal IX/X motor nuclei, the intermediate reticular zone, the medulla oblongata, the pontine tegmentum, the caudal RN, the gigantocellular reticular nucleus, the coeruleus–subcoeruleus complex, the pars compacta of the substantia nigra, the basal prosencephalon, the mesocortex, and the neocortex. However, multiple lines of evidence suggest that LP is not systematically seen in the PD brain and LP is also documented in healthy individuals (24). Also, in some cases of PD, and most notably in early-onset genetic forms, loss of SNc DA neurons has been reported to occur in the absence of detectable LP (25–27).

Although the role of LP in the pathogenesis of PD has been the subject of much debate (28), the detection of LP has remained central in investigations of the key brain regions and circuits underlying PD pathophysiology. In this context, it may be useful to focus attention on brain and PNS regions that show documented cell death and/or axonal degeneration, irrespective of the presence or absence of LP. This could perhaps provide new perspectives on the actual, more proximate, causes of the major symptoms of the disease and their progression. Relevant to the present point, in their most recent and insightful work, Braak and Tredici write, “We ascribed the same weight to axonopathy and nerve cell dysfunction (presumably attributable, but not limited, to the presence of Lewy pathology) as to neuronal death because the development of pathology together with neurotransmitter loss, axonal, and somatodendritic dysfunction in multiple neuronal populations could prove to be more stressful for involved neurons over time than premature cell death within a select neuronal population” (6).

## Where and when does neuronal loss appear in PD?

Loss of neurons in the brain is thought to occur in the context of normal aging. For example, there have been multiple publications reporting significant age-dependent decline in neuron number in the SNc (29–37), as well as in regions such as the PPN (38), and LC (39, 40). Above and beyond such cell loss associated with normal aging, a key question is where in the brain can one find substantial neuronal loss in PD?

Although numerous publications have referred to cell loss occurring in many CNS and PNS regions in the context of PD, we believed it germane to re-evaluate the published scientific literature addressing this question.

To do so, we took great care to find work concentrating on neuronal loss and not only denervation [as is common for the heart, for example (41–43)]. We found 90 primary research articles reporting PD-specific cell loss in the following regions (Table 1): the SNc, VTA, amygdala, cortex, DMV, hypothalamus, laterodorsal tegmental nucleus, LC, NBM, OB, oral pontine reticular nucleus, PPN, pre-supplementary motor cortex, RN, supraoptic nucleus, sympathetic/parasympathetic ganglia, and thalamus. These original articles span from 1953 to 2015. The techniques used to quantify cell loss varied, and we have classified them accordingly. Across all regions examined, 14 of the examinations were defined as *observational*, 39 as implicating *manual* counting, 18 used *computer-assisted* counting, and 26 used *stereological* counting methods. While informative, the value of observational studies can be considered limited given their lack of precision and the fact that they are greatly influenced by the observer. Lack of bias is also difficult to assure in studies involving manual counting. This technique is also unable to assure that a cell is not being counted twice if present in two subsequent sections. Other techniques such as computer-assisted counting were developed to improve on these aforementioned methods, however, these are also limited in that they often lack rigorous systematic sampling, are sensitive to tissue shrinkage, and are often unable to account for local tissue thickness, or for cells damaged on slice edges. These issues are systematically addressed using modern stereological counting techniques. Another issue to consider is that many of the studies included in this review, including those employing stereology, either did not use age-matched controls, or did not state whether counting was conducted blind to diagnosis. Yet another apparent feature of this literature is the diversity of method iterations used, the varying number of brain regions assessed in each study and, importantly, the stage or type of PD studied (and how this was defined). Here, we will discuss the evidence of cell loss (if not otherwise stated, relative to healthy control cases), ordering the regions in subsections according to the strength of the evidence (Table 1).

**Table 1:** List of 90 studies quantifying the loss of neurons in the brain in PD.

**Regions**	**Publications (reference #)**	**Technique**	**N (ctrl)**	**Loss of neurons (%)**	**Comparison group info (healthy controls unless stated otherwise)**	**Blinded/age matched**	**Stated diagnosis, scale of severity, disease duration (expressed in range or mean, when available)**	**Other regions counted**	**Correlations (with disease severity, duration or age)**
Substantia nigra pars compacta (SNc)	Greenfield and Bosanquet (44)	o	19 (22)	Some	–	Not stated	iPA, <1–20 years	LC	–
	Pakkenberg and Brody (45)	m	10 (10)	66	Healthy controls and two young controls	Not stated/Yes	iPA	–	–
	Bernheimer et al. (46)	o	69 (0)	Some	No healthy controls, compared to type of PD and Huntington's disease	Not stated/Yes	PD, H&Y, 1–47 years	–	–
	Rajput and Rozdilsky (47)	o	6 (1)	Some	–	Not stated	iPA, H&Y, 3–18 years	LC, DMV, Cortex, Hypothalamus, Intermediolateral spinal cord, sympathetic ganglia	–
	Gaspar and Gray (48)	o	32 (6)	Some	–	Yes/Yes	iPD, 2–23 years	LC, NBM	–
	Tagliavini et al. (49)	o	6 (5)	Some	–	Not stated/Yes	iPD, 5–13 years	NBM	–
	Chan-Palay (50)	o	9 (22)	Some	–	Yes/Not stated	PD	NBM	–
	Gibb and Lees (51)	m	34 (–)	–	No healthy controls, compared young and old onset	Not stated	PD, 1–34 years	–	–
	Hirsch et al. (52)	c	4 (3)	77	–	Not stated	PD	A10, A8, CGS	–
	German et al. (53)	c	5 (3)	61	–	Not stated/Yes	PD, 5–27 years	VTA	–
	Rinne et al. (54)	s	12 (18)	60	–	Not stated/Yes	iPD, H&Y II–V	–	Yes
	Zweig et al. (55)	o	6 (8)	Mild to severe	Not compared—estimation	Not stated/Yes	PD, 5–14 years	PPN, DR, NBM	–
	Gibb et al. (56)	m	6 (6)	75	–	Not stated	PD	–	–
	Halliday et al. (57)	c	4 (4)	68	–	Not stated/Yes	PD	SNc + LC, RN, PPN, DMV	Yes (dementia score)
	Fearnley and Lees (31)	m	20 (36)	20–90	–	Not stated/Yes	PD, 1.5–38 years	–	Yes (also in controls)
	Pakkenberg et al. (58)	s	7 (7)	66	–	Not stated/Yes	PD, 4–16 years	–	–
	Paulus and Jellinger (59)	m	39 (14)	59	–	Not stated/Yes	PD, H&Y III–V, 1–31 years	LC, DRN, NBM	–
	Xuereb et al. (60)	o	5 (5)	Some	–	Not stated/Yes	PD	Thalamus (multiple nuclei)	–
	Moller (61)	c	3 (3)	80	–	Not stated/Yes	PD	–	–
	Zweig et al. (62)	m	13 (14)	Some	–	Yes	PD, H&Y 4.5, 11 years	LC, VTA, NBM	–
	Mouatt-Prigent et al. (63)	c	4 (3)	76	–	Not stated/Yes	iPD	VTA	–
	Ma et al. (64)	s	4 (7)	70	–	Not stated	PD	–	–
	Halliday et al. (65)	s	11 (15)	37–75	–	Not stated/Yes	PD, 1–18 years	–	Yes
	Ma et al. (66)	c	20 (8)	76	–	Not stated/Yes	PD	–	–
	Ma et al. (67)	s	12 (12)	55	–	Not stated/Yes	PD, H&Y III–V, 3–17 years	–	Yes
	Damier et al. (68)	c	5 (5)	86	–	Not stated	iPD	VTA	Yes
	Henderson et al. (69)	c	9 (8)	69	–	Not stated/Yes	PD, H&Y II–V, 3–17 years	Centromedian–Parafascicular Complex, mediodorsal or anterior principal nucleus	–
	Zarrow et al. (70)	m	19 (13)	78	Healthy controls, AD	Not stated/Yes	iPD, 12.4 years	LC, NBM	
	Greffard et al. (71)	o	14 (5)	50	–	Not stated/Yes	iPD, UPDRS3 = 53, 8.5 years	–	Yes
	Rudow et al. (35)	s	8 (23)	~80 vs. young, ~75 vs. old controls	Young, middle aged and old healthy controls	Not stated/Yes	PD, 7–20 years	–.	Yes, in controls
	Beach et al. (72)	o	66 (87)	some	Healthy controls, ILDB, DLB, ADLB, ADNLB	Yes/Not stated	PD + DLB, UPDRS = 41, 10.6 years	–	–
	Karachi et al. (73)	s	12 (8)	69–88	–	Yes	PD, UPDRS	PPN	–
	Milber et al. (74)	s	13 (17)	70	Healthy controls, iLBD	Yes/Not stated	PD, Braak stage I–VI, 8.3 years.	–	Yes in iLBD
	Kordower et al. (75)	s	28 (9)	50–90	–	Yes	PD, 1–27 years	–	Yes
	Dijkstra et al. (76)	s	24 (12)	56	Healthy controls, iLBD	Yes	PD and iLBD, Braak stage 0–VI, H&Y, 13.6 years	–	Yes
	Kraemmer et al. (77)	m	4 (0)	–	No healthy controls, compare to AD, CJD, CBS, NPH	Yes/Not stated	PD and DLB, 2–4 years	–	–
	Cheshire et al. (78)	s	44 (17)	75	–	Yes	PD, LID severity, 14.8 years	RN	–
	Iacono et al. (79)	s	6 (6)	82	–	Yes	iPD and iLDB, Braak stage I–IV, H&Y 2–5,	–	–
Total	**38**	**o10, m8, c8, s12**	**612 (452)**						
Locus coeruleus (LC)	Rajput and Rozdilsky (47)	o	6 (1)	Some	–	Not stated	iPA H&Y, 3–18 years	SN, DMV, Cortex, Hypothalamus, Intermediolateral spinal cord, sympathetic ganglia	–
	Gaspar and Gray (48)	o	32 (6)	Some	–	Yes	iPD, 2–23 years	SNc, NBM	–
	Hirsch et al. (52)	c	4 (3)	55	–	Not stated	PD	SNc, A10, A8	–
	Chan-Palay and Asan (80)	c	6 (3)	31–94*	–	Not stated/Yes	PD	–	–
	Zweig et al. (55)	o	6 (8)	Mild to severe	Not compared—estimation	Not stated/Yes	PD, 5–14 years	PPN, SNc, DR, NBM	–
	Halliday et al. (57)	c	4 (4)	68	–	Not stated/Yes	PD	SNc + LC, RN, PPN, DMV	–
	Gai et al. (81)	c	6 (5)	74	–	Not stated/Yes	iPD, 5–30 years	PPN, LTN, OPN, RN	Yes
	Paulus and Jellinger (59)	m	37 (12)	63	–	Not stated/Yes	PD, H&Y III–V, 1–31 years	SNc, DRN, NBM	–
	German et al. (82)	c	6 (7)	21–93	Healthy controls, AD, down-syndrome	Not stated/Yes	PD, 5–16 years	–	–
	Patt and Gerhard (83)	o	8 (8)	Some	–	Not stated	PD	–	–
	Zweig et al. (62)	m	13 (14)	46–69	–	Yes/Yes	PD, H&Y 4.5, 11 years	SNc, VTA, NBM	–
	Hoogendijk et al. (84)	c	5 (5)	39 NS	Healthy controls, AD, ALS	Not stated/Yes	PD, 7 years	–	–
	Bertrand et al. (85)	c	11 (6)	58–78	–	Not stated	PD	–	Yes
	Zarrow et al. (70)	m	19 (13)	83	Healthy controls, AD	Not stated/Yes	iPD, 12.4 years	SNc, NBM	–
	Brunnstrom et al. (86)	m	25 (0)	Mild-severe	Healthy controls, AD	Yes/Not stated	DLB and PD dementia	–	–
	McMillan et al. (87)	m	7 (8)	71–88	Healthy controls, AD, DLB	Yes	PD, 7–25 years	–	–
	Dugger et al. (88)	c	21 (11)	Some	–	Not stated/Yes	LBD, 8.4 years	PPN	–
	Del Tredici and Braak (89)	o	5 (1)	Some	–	Not stated	PD, H&Y 3–5, 7–15 years	–	–
Total	**18**	**o5, m4, c9, s0**	**221 (115)**						–
*31 w/o dementia, 48 w/dementia, 94 if Non-responsive to L-dopa									
Nucleus basalis of meynert (NBM)	Arendt et al. (90)	m	5 (14)	70	–	Not stated/Yes	Postencephalitic PD	–	–
	Candy et al. (91)	m	5 (5)	Some	Healthy controls, AD	Not stated	PD	–	–
	Nakano and Hirano (92)	m	2 (5)	90	–	Not stated/Yes	PD-dementia complex of Guam, 4–5 years	–	–
	Whitehouse et al. (93)	m	12 (10)	45–71	–	Yes	iPD, 4–26 years	–	–
	Gaspar and Gray (48)	m	32 (6)	36	–	Yes	iPD, 2–23 years	SNc, LC	–
	Nakano and Hirano (94)	m	11 (13)	60	–	Not stated/Yes	PD, 1–17 years	–	–
	Tagliavini et al. (49)	m	6 (5)	46–69	–	Not stated/Yes	iPD, 5–13 years	SNc	–
	Perry et al. (95)	m	4 (8)	17–72	Healthy controls, AD	Not stated/Yes	PD	–	–
	Rogers et al. (96)	m	4 (5)	Some	Healthy controls, PSP, Creutzfeldt-Jakob disease, ALS, MS and AD (+ individual cases of other diseases)	Not stated/Yes	PD	–	–
	Chan-Palay (50)	m	9 (22)	~50	Healthy controls, AD	Yes/Not stated	PD	SNc	–
	Paulus and Jellinger (59)	m	40 (17)	Some	–	Not stated/Yes	PD, H&Y III–V, 1–31 years	SNc, LC, DRN	–
	Zweig et al. (62)	o	13 (14)	Some	–	Yes	PD, H&Y 4.5, 11 years	LC, SNc, VTA	–
	Zarrow et al. (70)	m	19 (13)	37	Healthy controls, AD	Not stated/Yes	iPD, 12.4 years	SNc, LC	–
Total	**13**	**o1, m12, c0, s0**	**162 (137)**						
Pedunculopontine nucleus (PPN)	Hirsch et al. (97)	c	6 (4)	57	Healthy controls, supranuclear palsy	Not stated	PD	–	–
	Jellinger (98)	m	14 (15)	53	–	Not stated/Yes	PD, 10 years	–	–
	Zweig et al. (55)	m	4 (8)	46–69	–	Not stated/Yes	PD, 10–14 years	–	–
	Halliday et al. (57)	c	4 (4)	57	–	Not stated/Yes	PD	SNc + LC, RN, DMV	–
	Gai et al. (81)	c	6 (5)	43	–	Not stated/Yes	iPD, 5–30 years	LTN, OPN, RN, LC	Yes
	Rinne et al. (99)	s	11 (9)	40	–	Not stated/Yes	PD, H&Y 2.5 and 5, 9.3 years	–	Yes
	Schmeichel et al. (100)	m	13 (11)	65	Healthy controls, MSA	Yes/Not stated	DLB, 3–16 years	Laterodorsal tegmental nucleus	–
	Karachi et al. (73)	s	12 (8)	31–38	–	Yes	PD, UPDRS 0–IV	SN	–
	Dugger et al. (88)	c	21 (11)	Some	–	Not stated/Yes	LBD, 8.4 years	LC	–
	Hepp et al. (101)	s	9 (9)	41	Healthy controls, DLB	Yes	PD, Braak stage IV–VI, H&Y IV–V, 8–26 years	–	–
	Pienaar et al. (102)	s	8 (5)	50	–	Yes	PD, Braak stage II–IV, 6–13 years	–	–
Total	**11**	**o0, m3, c4, s4**	**108 (89)**						
Hypothalamus	Rajput and Rozdilsky (47)	o	6 (1)	None	–	Not stated	iPA, H&Y, 3–18 years	SN, LC, DMV, Cortex, intermediolateral spinal cord, sympathetic ganglia	–
	Kremer (103)	m	8 (15)	None	–	Not stated	PD	–	–
	Kremer and Bots (104)	m	8 (7)	None	–	Not stated/Yes	iPD, 4–17 years	–	–
	Purba et al. (105)	m	6 (6)	20	–	Not stated/Yes	PD	–	–
	Nakamura et al. (106)	m	8 (6)	None	–	Not stated/Yes	iPD	–	–
	Ansorge et al. (107)	m	7 (8)	12–29	–	Not stated/Yes	PD, 18 years	–	–
	Hoogendijk et al. (108)	m	12 (6)	None	–	Yes	iPD	–	–
	Fronczek et al. (109)	c	9 (9)	45	–	Yes	PD, late-stage	–	–
	Thannickal et al. (110)	s	10 (5)	50	–	Not stated/Yes	PD, H&Y I–V, 4–23 years	–	Yes
Total	**9**	**o1, m6, c1, s1**	**74 (63)**						
Dorsal motor nucleus of the vagus nerve (DMV)	Eadie (111)	m	8 (5)	30	–	Not stated/Yes	PD	Hypoglossal nuclei, nucleus ambiguus	–
	Rajput and Rozdilsky (47)	o	6 (1)	Some	–	Not stated	iPA, H&Y, 3–18 years	SN, LC, Cortex, Hypothalamus, Intermediolateral spinal cord, sympathetic ganglia	–
	Halliday et al. (112)	c	4 (4)	77	–	Not stated	PD	RN	–
	Halliday et al. (57)	c	4 (4)	77	–	Not stated/Yes	PD	SNc + LC, RN, PPN	–
	Saper et al. (113)	m	5 (5)	60	–	Not stated	PD, 2–16 years	–	–
	Gai et al. (114)	s	8 (6)	55	–	Not stated/Yes	PD, 5–24 years	Hypoglossal nucleus	Yes
	Benarroch et al. (115)	o	14 (12)	50	–	Yes/Not stated	PD or LBD, 10 years	Nucleus ambiguus	–
Total:	**7**	**o2, m2, c2, s1**	**49 (37)**						
Raphe nuclei (RN)	Yamamoto and Hirano (116)	m	2 (1)	50–90	–	Not stated/Yes	iPD	–	–
	Halliday et al. (112)	c	4 (4)	0 dorsal-56 median	–	Not stated	PD	DMV	–
	Halliday et al. (57)	c	4 (4)	0 dorsal-44 obscurus-60 median	–	Not stated/Yes	PD	SNc + LC, PPN, DMV	–
	Gai et al. (81)	c	6 (5)	76	–	Not stated/Yes	iPD, 5–30 years	PPN, LTN, OPN, LC	–
	Paulus and Jellinger (59)	m	23 (6)	37	–	Not stated/Yes	PD, H&Y III–V, 1–31 years	SNc, LC, RN, NBM	–
	Benarroch et al. (117)	m	14 (12)	60–67	–	Yes	DLB, 5–20 years	–	–
	Cheshire et al. (78)	s	44 (17)	None	–	Yes	PD, LID severity, 14.8 years	SNc	–
Total:	**7**	**o0, m3, c3, s1**	**97 (49)**						
Ventral Tegmental Area (VTA)	Javoy-Agid et al. (118)	m	2 (2)	77	–	Not stated	PD	–	–
	Hirsch et al. (52)	c	4 (3)	48	–	Not stated	PD	SNc, A10, A8, CGS	–
	German et al. (53)	c	5 (3)	42	–	Not stated/Yes	PD, 5–27 years	SNc	–
	Zweig et al. (62)	m	13 (14)	Some	–	Yes	PD, H&Y 4.5, 11 years	LC, SNc, NBM	–
	Mouatt-Prigent et al. (63)	c	4 (3)	Some	–	Not stated/Yes	iPD	SNc	–
	Dymecki et al. (119)	m	7 (6)	41–62	–	Not stated/Yes	PD, long-term	–	–
	McRitchie et al. (120)	s	3 (3)	31	–	Not stated/Yes	iPD, 1–27 years	A8, A10	–
	Damier et al. (68)	c	5 (5)	46	–	Not stated	iPD	SNc	Yes
Total	**8**	**o0, m3, c4, s1**	**43 (39)**						
Olfactory bulb (OB)	Pearce et al. (121)	m	7 (7)	57	–	Not stated/Yes	PD, 8–19 years	–	–
	Huisman et al. (122)	s	10 (10)	Increase of 100	–	Not stated/Yes	PD, 4–23 years	–	–
	Huisman et al. (123)	s	20 (19)	Increase of 100 in female	–	Yes	iPD, 3–30 years	–	–
	Mundinano et al. (124)	s	6 (15)	Increase Of 100	–	Not stated/Yes	PD, Braak stage II–V	–	–
Total	**4**	**o0, m1, c0, s3**	**43 (51)**						
Thalamus	Xuereb et al. (60)	m	5 (5)	None	–	Not stated/Yes	PD	Thalamus (multiple nuclei)	–
	Henderson et al. (125)	c	9 (10)	40–55	–	Not stated/Yes	PD, H&Y II–V, 7.2 years	Caudal intralaminar nuclei, limbic thalamic nuclei	–
	Henderson et al. (69)	s	9 (8)	50–70	–	Not stated/Yes	PD, H&Y II–V, 3–17 years	SNc, Centromedian–parafascicular complex, mediodorsal or anterior principal nucleus	–
	Halliday et al. (126)	s	9 (9)	None	–	Not stated/Yes	PD, H&Y II–V, 9 years	Motor thalamus, Cortex	–
Total	**4**	**o0, m1, c1, s2**	**32 (32)**						
Sympathic/parasympathic ganglia	Rajput and Rozdilsky (47)	o	6 (1)	Some	–	Not stated	iPA, H&Y, 3–18 years	SN, LC, DMV, Cortex, Hypothalamus	–
	Wakabayashi and Takahashi (127)	m	25 (25)	31–43	–	Not stated/Yes	PD	–	–
	Benarroch et al. (115)	o	14 (12)	None	–	Yes/Not stated	PD or LBD, 10 years	DMV, nucleus ambiguus	–
Total:	**3**	**o2, m1, c0, s0**	**45 (38)**						
Cortex	Rajput and Rozdilsky (47)	o	6 (1)	None	–	Not stated	iPA, H&Y, 3–18 years	SN, LC, DMV, Hypothalamus, Intermediolateral spinal cord, sympathetic ganglia	–
	Pedersen et al. (128)	s	10 (12)	None	–	Not stated/Yes	PD, 2–25 years	–	–
Total	**2**	**o1, m0, c0, s1**	**16 (13)**						
Pre-supplementary and premotor cortex	MacDonald and Halliday (129)	m	5 (5)	32–45	–	Yes	PD, 10–17 years	–	–
	Halliday et al. (126)	s	9 (9)	None	–	Not stated/Yes	PD, H&Y II–V, 9 years	Motor thalamus	–
Total	**2**	**o0, m1, c0, s1**	**14 (14)**						
Amygdala, corticomedial complex	Harding et al. (130)	s	**18 (16)**	30	–	Yes	PD, 13 years	–	–
Hippocampus	Joelving et al. (131)	s	**8 (8)**	None	–	Not stated/Yes	PD, 2–25 years	–	–
Laterodorsal tegmental nucleus (LTN)	Gai et al. (81)	c	**6 (5)**	41	–	Not stated/Yes	iPD, 5–30 years	PPN, OPN, RN, LC	Yes
Oral pontine reticular nucleus (OPN)	Gai et al. (81)	c	**6 (5)**	41	–	Not stated/Yes	iPD, 5–30 years	PPN, LTN, RN, LC	Yes

Included in the table are the technique used for quantification (o, observation; m, manual c, computer assisted; s, stereological counting), the number of subjects and controls (ctrl) studied, the estimated % loss of neurons, any particularity in the comparison group, mention if studies were performed blind and with age-matched controls, the stated diagnosis, scale of severity and disease duration when mentioned and note on other regions counted. Where an average value of loss was not given by authors, this number was calculated from available data. Bold values indicates total numbers per region. *Indicates details which are given at the end of that section.

## Substantia nigra pars compacta

Loss of SNc DA neurons in PD is indisputable. Here we found 38 studies addressing this directly with a total of 612 brains. However, if we consider the methods used, we found that 10 of these studies were observational, 8 involved manual counting methods, 8 used computer-assisted methods, and 12 used stereology. Considering stereological methods as best practice for unbiased evaluation of cell number, 181 brains were quantified as such for SNc: still a large number. The average cell loss reported for studies involving stereological methods is ~68%. The definition and clinical stage of PD in most studies varied greatly, especially in reporting. For example, for the 12 studies using stereological methods, three papers (74, 76, 79) staged each case according to the Braak staging (to be expected given that Braak staging only came about in the early 2000s). In the same 12 studies, the age “since disease onset” varied between 1 and 27 years when stated, the Hoehn and Yahr ratings (H&Y, used to describe the progression severity of PD symptoms) varied between 2 to 5 and the UPDSR score (that includes H&Y rating, symptoms and quality-of-life scores) was also on occasion provided. A correlation with disease duration/severity was found in 10 studies. It is relevant here to mention that some authors, including Gibb et al. (56) have discussed the selective vulnerability of restricted sub-regions within the SNc. These data are important and relevant to the progression of the field; however, we found this distinction absent in the majority of the work we examined.

**Methodology and Scales of PD Progression**

We searched the scientific literature using the search engines and databases of PubMed, Google Scholar and Science Direct. The following search terms were used: “PD,” and “cell loss,” “cell death,” or “reduced cell/neuron number.” Furthermore, these terms were used in combination with brain structure keywords: “SNc,” “VTA,” “LC,” “Raphe,” “DMV,” “PPN,” “NBM,” and “enteric system” (“ENS”), and “gut.” Review and original article abstracts were screened, then, where appropriate, read. Where any direct or indirect claim for cell loss was found (rather than only the presence of LP), the claim was followed to its original source.

The Hoehn and Yahr scale (H&Y) is a widely used clinical rating scale, which defines broad categories of motor function in PD (where 1 is the least severe, and 5, most severe symptoms) (132).

Braak staging is a method of classifying the progression of PD pathology and symptoms based on the presence of Lewy pathology (where 1 represents initial pathology in the brain stem, and 6, severe pathology including the neocortex) (21).

## Pedunculopontine nucleus and locus coeruleus

The evidence for cell loss for both the PPN (11 studies), containing cholinergic neurons and the LC (18 studies), containing noradrenergic neurons, is also relatively strong.

For the PPN, four studies used stereological methods. In these four studies, the average loss of cholinergic PPN neurons was 41% and the range of PD stages amongst the subjects evaluated was broad. For example, in Rinne et al. (99), the PD cases ranged from a H&Y rating of 2.5 to 5; in Karachi et al. (73), UDPRS score was used, and in both Hepp et al. (101), and Pienaar et al. (102), the PD cases were between Braak stages 4 and 6 and between 2 and 4, respectively. Although sample sizes were relatively small in these two last studies, nine and eight, respectively, it is somewhat surprising that in the most advanced PD group, loss of cholinergic PPN neurons was not higher than for less advanced PD subjects, contrarily to the report by Rinne et al. (99).

Surprisingly, we found no study quantifying loss of LC neurons using stereological counting methods. For the LC, 221 brains were studied, with cell loss ranging from “some” to 94%. Five of the studies were based on observational quantifications, 4 on manual counting and 9 used computer-assisted counting. In these 18 papers, when stated, the H&Y score was between 3 and 5, and disease duration was between 1 and 31 years. A correlation of the extent of cell loss with disease duration was found in two of these studies (81, 85).

## Dorsal motor nucleus of the vagus, raphe nuclei, nucleus basalis of meynert and ventral tegmental area

Substantial cell loss has been documented in the DMV, containing cholinergic neurons, with 7 studies evaluating this loss in 49 cases. Of these, only one study (114) used stereology, where they reported 55% neuronal loss in eight PD cases, ranging from 5 to 24 years post-diagnosis and reported correlation with disease duration/severity.

The importance of re-evaluating cell loss is PD is apparent when considering the serotonergic RN. For these nuclei, which are considered by many authors to be lost in PD, we found 7 papers describing neuronal loss varying between 0 to 90%. Cheshire et al. however, using stereology in 44 late-stage PD subjects, found no cell loss in the dorsal raphe nucleus (78). In the NBM, containing cholinergic neurons, we found 13 papers, 12 using manual counting methods, and one observational, which estimated an average neuronal loss of between “some” to 72%. No correlation with disease duration was reported. The high prevalence of concomitant PD and Alzheimer's disease (AD) might explain why cell loss varied so much for this region. Surprisingly, only 8 studies directly evaluated neuronal loss in the VTA, a dopaminergic region often considered to be only modestly affected in PD. Of these, one study used stereology (120) to evaluate the loss of neurons in 3 cases of PD (or 6 including PD with a secondary diagnosis) that were between 1 and 27 years post-diagnosis and reported an average neuronal loss of 31%. One paper reported correlation of the extent of cell loss with disease duration (68).

## Thalamus, hypothalamus, olfactory bulb

Four studies reported neuronal loss in thalamic nuclei, with 2 using stereology (69, 126). In (69), 9 subjects with H&Y disease ratings between 2 and 5 statistically significant loss of 30–40% was reported in the centromedian-parafasicular complex. However, no loss was found in the motor thalamus in 9 subjects with similar H&Y disease ratings in the work of Halliday et al. (126). Neuronal loss has also sometimes been reported in the hypothalamus (9 studies), with one using stereology; Thannickal et al. (110) reported a 50% cell loss in 10 PD cases, with increased loss with disease severity. Olfactory dysfunction is now well established as an early symptom of PD. Four studies evaluating cell loss in the olfactory bulb were reported. One of these (121) described a 57% decrease in neuronal number (identified as cells with “a prominent nucleolus surrounded by Nissl substance”), while the others (122–124), using stereology, reported a 100% increase in the number of TH-positive neurons.

## Peripheral nervous system, spinal cord and other brain regions

Though there is substantial evidence for LP occurring in the ENS (133), we did not find any study reporting direct—quantitative evidence—for neuronal loss in the gut. Though it has been inferred that ENS glial cell loss is occurring (134), there is evidence that neuronal loss in the gut is not associated with PD (135). Of note, a publication often cited in support of neuronal loss in the ENS (115) shows, in fact, neuronal loss in the DMV. With regards to the spinal cord, published evidence is also scarce; of the studies most relevant here, Wakabayashi et al. (127), using manual counting methods, described a loss of 31% and 43% respectively in the 2nd and 9th thoracic segments of the intermediolateral of the spinal cord. For the amygdala, the pre-supplementary motor cortex, several other cortical regions, the laterodorsal tegmental nucleus and the oral pontine reticular nucleus, we found only single studies supporting loss, with stereology used for the amygdala (30% loss) (130), and cortex (10% loss) (130) (see Table 1).

## Regional order of cell loss?

In summary, it seems clear that there is some level of cell loss in PD in restricted regions including the SNc, LC, NBM, PPN, DMV, VTA, and probably the RN. However—because of the lack of data for some regions, the variety of techniques used to count neurons, potentially numerous unintentional sources of bias, and because of the inconsistency in criteria used for subject sampling—firm conclusions are somewhat limited. In particular, it is difficult to conclude on the relative extent and temporal order of cell loss in these different brain regions as a function of disease progression, information that would be critical to advance the field. Indeed, a direct comparison of the extent of neuronal loss in different regions examined in different studies is hazardous, even if stereological studies were to be selected. Interestingly, of the 38 studies we identified evaluating cell loss in the SNc, only 5 of these also looked at the VTA, and of these only 1 used stereology. Given the importance of the difference in vulnerability of these two nuclei, a systematic evaluation of the extent of loss of these neurons in PD would be very informative. But even if as a technique, stereology mitigates for most of the classic biases, it is still unable to account for the variation in subject sampling, i.e., variation in disease duration, sex and age, unless these criteria were considered in a similar way for each study. Unfortunately, this has not, thus far, been the case. In conclusion, it seems clear that stereological studies comparing multiple regions in the same subjects and these regions in subjects at different stages of PD are critically needed to advance the field.

## What are the common features shared by neurons affected in PD?

**Figure 1:**
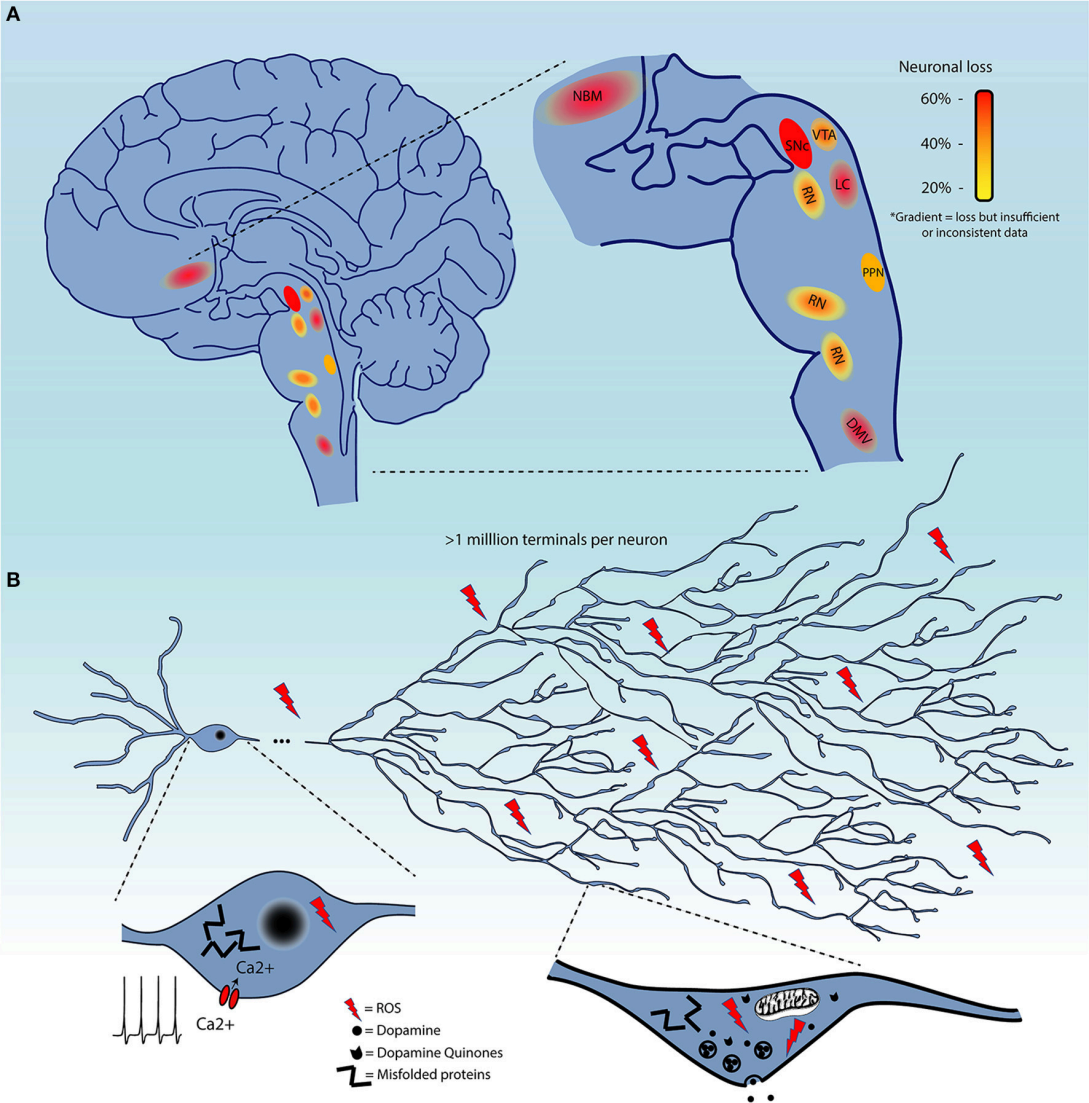
**(A)** Schematic representation of brain regions demonstrating cell loss in Parkinson's disease. These are color-coded based on the evidence of cell loss. Red = 60%, orange = 40%, and yellow = 20%. Color gradients indicate uncertainty in the extent of this cell loss. **(B)** Summary of the converging hypotheses that may explain the origins of the selective vulnerability of neurons in Parkinson's disease. This includes the exceptionally large axonal arbor of PD-affected neurons, their electrophysiological properties, including calcium-dependent pacemaking, and high levels of oxidant stress in the somatodendritic and axonal domain, all thought to be contributing to cellular dysfunction and cell loss. Pathological protein aggregation and reactive dopamine quinones are considered as additional precipitating factors.

Although, as mentioned previously, the evidence for the extent of cell loss in regions other than the SNc in the PD brain is not always sufficiently documented, it is clear that some level of cell loss occurs in a limited subset of regions beyond the SNc (Figure 1A), or, to the least, that neuronal functions including neurotransmission are perturbed in multiple neuronal circuits. It is therefore of great interest to identify some of the biological features that distinguish neuronal subgroups in terms of their basal vulnerability to some of the cellular stresses that are invoked to trigger PD, including altered proteostasis (due to lysosomal and/or proteosomal impairment), mitochondrial dysfunction, and sustained oxidant stress (including from highly reactive DA metabolites).

Several groups have been tackling this question by interrogating the characteristics that render neurons, starting with those of the SNc, particularly vulnerable to degeneration / cell death (136–138). It is likely that some shared functional or structural properties are responsible for selective vulnerability of affected nuclei, as opposed to features truly unique to SNc DA neurons. The causative characteristic(s) should be present in all affected neurons, but also be absent in neurons that do not degenerate or that degenerate much later in the disease. Four main converging hypotheses on selective vulnerability in PD have been gaining attention lately (Figure 1B), related to DA toxicity, iron-content, autonomous pacemaking and axonal arborization size. The next section will explore the likelihood that these hypotheses can explain why select neuronal populations are particularly vulnerable in PD.

## Dopamine toxicity

Firstly, it has been suggested that DA neurons in general are most at risk because they produce DA as a neurotransmitter, a molecule that can be toxic in certain conditions through the generation of reactive quinones during its oxidation (139). This oxidation has been proposed to be implicated in the production of neuromelanin in SNc DA neurons. These DA quinones have been shown to interact with and negatively impact the function of mitochondrial protein complexes I, III, and V (140) and of other proteins such as tyrosine hydroxylase, the DA transporter and α-synuclein (141, 142). Such reactive by-products can promote mitochondrial dysfunction, pathological aggregation of proteins such as α-synuclein and oxidative stress (143). Increasing the vesicular packaging of DA accordingly reduces the vulnerability of DA neurons, while down-regulating vesicular packaging has the opposite effect (144–147). Although highly relevant, this phenomenon alone does not readily explain the differential vulnerability of different dopaminergic neuron subgroups (such as SNc vs. VTA) and cannot contribute to the potential vulnerability of non-dopaminergic neurons in PD. Also, in the context of DA-induced toxicity, it is puzzling that levodopa therapy, acting to increase DA synthesis, does not appear to accelerate cell loss (148, 149). For these reasons, even if DA toxicity most certainly contributes to degeneration of SNc DA neurons, it is certainly not the sole factor driving neuronal death in PD.

## Iron content

Secondly, iron content is thought to also be an important contributor to the selective vulnerability of SNc DA neurons. Iron is known to be able to generate ROS by the Fenton reaction and has been shown to accumulate with age in SNc (150–152). Since the mitochondrial electron transport chain relies on iron sulfur clusters for its function and since it is believed that SNc neurons have particularly high bioenergetic demands (136, 138, 153), elevated iron content could in part underlie elevated and sustained mitochondrial activity. Another interesting feature of iron in SNc DA neurons is that it can be chelated by neuromelanin, which renders it unavailable for mitochondrial function. Even if the affinity of iron for neuromelanin is much lower than for other iron binding proteins such as ferritin, it is possible that accumulation of neuromelanin and loss of ferritin concentration with age impacts gradually mitochondrial function, which could eventually promote cell death. However, data about potential iron content and iron-binding protein concentration changes in PD is still a matter of debate (154, 155). In addition, data is lacking on iron levels in other brain regions presenting cell death in PD. In fact, the only other region studied in this context has been the LC, which did not show high iron relative to the SNc (156–159).

## Autonomous pacemaking

A third highly attractive hypothesis to explain the vulnerability of SNc DA neurons has its origins in the fact that these neurons demonstrate autonomous pacemaking. Many receptors/channels can potentially modulate the excitability and survival of DA neurons (160). The fact that pacemaking activity in SNc DA neurons is accompanied by slow oscillations in intracellular calcium concentrations, caused by the opening of voltage-dependent Cav1 plasma membrane calcium channels (Cav1.1 and 1.3) has recently renewed interest to this topic. In the Cav1 family, Cav1.3 has been suggested to be of particular interest because its voltage-sensitivity and inactivation properties allow a subset of the calcium channels to always stay open during pacemaking, causing extensive calcium entry (137). These oscillations have a positive contribution to cell physiology because they help maintain pacemaking and directly promote mitochondrial oxidative phosphorylation (OXPHOS) (161). However, by doing so, they have been proposed to also promote chronically high levels of ROS production (162, 163). Along with a reduction in mitochondrial function with age, chronically elevated oxidative stress has been proposed to be a causative factor in the decline of neuronal survival (164). Interestingly, CaV currents and autonomous pacemaking are also a feature of LC and DMV neurons (162, 163), and have been hypothesized to be involved in their vulnerability. The fact that other neuronal populations also expressing Cav1.3 such as hippocampal neurons (165) and striatal spiny projection neurons (166) do not degenerate in PD highlights the possibility that the particular vulnerability of SNc DA neurons is due to a combination of physiological phenotypes and not only intracellular calcium oscillations. Intriguingly, recent post-mortem studies showed that there was no decrease in Cav1.3 mRNA level in early or late stage PD in human SNc compared to controls (166, 167), despite significant loss of SNc neurons. Finally, in addition to CaV channels, ATP sensitive potassium channels (K-ATP) have also been reported to regulate the excitability and vulnerability of SNc DA neurons (168).

## Axonal arborization size

A fourth hypothesis proposes that neurons such as those of the SNc are particularly vulnerable because of the massive scale of their axonal arborization, leading to very high numbers of axon terminals, elevated energetic requirements, and chronically high oxidant stress. Indeed, it has been shown that SNc DA neurons have an exuberant and highly arborized axonal arborization with estimates upwards of a million neurotransmitter release sites per SNc DA neuron in humans (136, 169): this would make them some of the most highly arborized neurons in the nervous system. This characteristic has the potential to place a very large bioenergetic burden on these cells, leaving little margin for additional bioenergetic stress (136, 138, 153). Related to this, it has been calculated that the ATP requirement for propagation of one action potential grows exponentially with the level of branching (170). In a recent publication (138), we demonstrated *in vitro* that reducing the axonal arbor size of SNc DA neurons to a size more similar to that of VTA DA neurons using the axonal guidance factor Semaphorin 7A, was sufficient to greatly reduce basal OXPHOS and reduce their vulnerability to toxins including MPP+ and rotenone. Although as previously discussed, the extent of neuronal loss is still unclear for many neuronal populations, it does seem likely that most neuronal nuclei affected in PD include neurons that are relatively few in number, but all possess long and profuse unmyelinated axonal arbors and a large number of axonal terminals (171–176). However, comparative data evaluating axonal arbor size amongst these populations and in populations of neurons that do not degenerate in PD is presently lacking. An interesting possible exception to this hypothesis could be striatal cholinergic interneurons, which were previously estimated in rats to present 500,000 axonal varicosities (177, 178), but have not been reported to degenerate in PD. This estimate was obtained by dividing the estimated number of terminals by the estimated number of cholinergic interneurons in the striatum, which was based on the total number of striatal neurons and the proportion of cholinergic interneurons. Considering recent stereological counting of the number of neurons in the rat striatum, it is possible that the total number of terminals estimated for striatal cholinergic neurons may have been overestimated by a factor of six (179). Based on this report, axonal arborization size of striatal cholinergic interneurons would be less than half of that of SNc neurons. Careful quantitative and comparative studies are clearly needed.

## A global bioenergetic failure hypothesis

One commonality between these four hypotheses is that they all suggest that vulnerable neurons are under intense mitochondrial/bioenergetic demand. This could alter the oxidative stress response by depleting antioxidants like glutathione (GSH), as previously suggested to occur in the PD brain (180–182). This stress could also, at a certain point, place the cells in a situation in which the rate of OXPHOS required to sustain neurotransmitter release and cellular excitability leaves too little of the cell's resources to sustain other key cellular functions such as degradation of damaged or misfolded proteins (137). This could lead to preferential dysregulation of axon terminals, triggering a dying back cascade culminating later in cell death (3, 183, 184). Approximately half of the oxygen consumed by mitochondria in SNc DA neurons appears to be used by activity-dependent cellular processes such as firing and neurotransmitter release (138). In this context, axon terminal degeneration seen early in the disease, prior to cell death, could be in part an attempt by stressed neurons to adapt to such excessively high metabolic needs. Such a dying back process could also lead to increased amounts of damaged axonal proteins to manage, potentially promoting their accumulation in intracellular inclusions. Since α-synuclein is highly concentrated in axon terminals, it is possible that retraction of axonal processes in a cell where protein degradation systems are overwhelmed, promotes creation of pathological aggregates of this protein, thus accelerating cell death. Interestingly, lysosomal defects secondary to GBA1 gene mutations are present in up to 10% of PD patients. This gene encodes a glucocerebrosidase responsible for breaking down lysosomal glucolipid. When GBA1 is mutated, the level of glucolipid and of misfolded proteins increases in neurons. This is likely to represent a particular challenge for highly arborized neurons such as those of the SNc, perhaps explaining why such mutations are now considered the greatest genetic risk factor for PD (185–191). Similarly, mutations in gene products implicated in mitophagy and mitochondrial antigen presentation (PARK2, PINK1) (192, 193), oxidative stress response (PARK7) (194, 195), or vesicular trafficking (LRRK2) (196, 197) are present in familial forms of PD and their detrimental impact on cellular functions could also represent larger challenges for highly arborized and energetically ambitious neurons.

## Toward better treatments of PD

In the context of the hypotheses discussed here regarding the origin of the selective vulnerability of neurons in PD, novel strategies to promote survival and preservation of cellular functions amongst challenged neuronal populations could possibly come from approaches that aim to reduce mitochondrial burden by either reducing neuronal metabolic needs or optimizing mitochondrial function. As an example, the CaV1.3 channel inhibitor isradipine is presently in phase 3 clinical trial and could possibly reduce the calcium- and activity-related metabolic stress of SNc DA neurons leading to neuroprotection (198). Other promising molecules could come from the repurposing of drugs used to treat diabetes and other metabolic diseases. One example is exenatide, a glucagon-like-peptide-1 agonist that has the property to increase glucose-induced insulin secretion, to prevent the rise of ROS and prevent decreases of mitochondrial function in diet-induced obese mice (199). This agonist was found to reduce the loss of DA neurons in the MPTP mouse model (200) and a recent clinical trial has shown improved motor function after 60 days of administration to PD patients (201). Overexpression of the mitochondrial deacetylase SIRT3 has also recently been shown in two studies to reduce basal OXPHOS by DA neurons and to protect SNc neurons in rodent models of PD (202, 203). With further discoveries of the underlying causes of the intrinsic vulnerability of neurons in the PD brain and PNS, multiple other strategies may soon be devised to address some of the specific challenges faced by energetically challenged neurons.

In conclusion, although the presently available data strongly argue that multiple populations of neurons are affected in PD and degenerate to varying extents, new work is needed to provide a more systematic, comparative, and time-dependent quantification of neuronal loss in this disease. More comprehensive and convincing data on cell death and axon terminal dysfunction in PD will likely provide additional impetus for new work aiming to solve the long-awaited challenge of identifying disease-modifying therapeutic approaches for this incapacitating and ill-treated disorder.

## Author contributions

NG and SB performed the litterature review. NG, SB, and L-ET wrote the manuscript. NG and SB contributed equally to this work.

### Conflict of interest statement

The authors declare that the research was conducted in the absence of any commercial or financial relationships that could be construed as a potential conflict of interest.

## Abbreviations

AD: Alzheimer Disease
ADLB: Alzheimer's Disease with Lewy bodies
ADNLB: Alzheimer's Disease with no Lewy bodies
ALS: Amyotrophic Lateral Sclerosis
CBS: corticobasal syndrome
CGS: central gray substance
CJD: Creutzfeldt-Jakob disease
ctrl: control
DLB: dementia with Lewy bodies
H&Y: Hoehn and Yahr scale
iPA: idiopathic paralysis agitans
LBD or iLBD: Lewy body disease or idiopathic Lewy body disease
LDB or iLDB: dementia with Lewy bodies or idiopathic dementia with Lewy bodies
LID: levodopa (L-dopa)–induced dyskinesias
MS: multiple sclerosis
MSA: multiple system atrophy
NPH: normal pressure hydrocephalus
PD or iPD: Parkinson's disease or idiopathic Parkinson's Disease
PSP: progressive supranuclear palsy
UPDRS: unified Parkinson disease rating scale.
